# Role of Oligomeric Proanthocyanidins Derived from an Extract of Persimmon Fruits in the Oxidative Stress-Related Aging Process

**DOI:** 10.3390/molecules19056707

**Published:** 2014-05-22

**Authors:** Takako Yokozawa, Chan Hum Park, Jeong Sook Noh, Seong Soo Roh

**Affiliations:** 1 Graduate School of Science and Engineering for Research, University of Toyama, 3190 Gofuku, Toyama 930-8555, Japan; 2 College of Korean Medicine, Daegu Haany University, Suseong-gu, Daegu 706-060, Korea; 3 Department of Food Science & Nutrition, Tongmyong University, Nam-gu, Busan 608-711, Korea

**Keywords:** oligomer, SAMP8, lifespan, stereotypical behavior, memory, VEGFR-2

## Abstract

Many researchers have focused on the oligomeric form of proanthocyanidins with a lower level of polymerization found in foodstuffs such as grape seeds and blackberries. The present study indicated that the oral administration of oligomers isolated from persimmon fruits extended the lifespan of senescence-accelerated mouse prone/8 (SAMP8), a murine model of accelerated senescence. On the other hand, oligomer-treated SAMP8 did not show stereotypical behavior. We also revealed that the oral administration of oligomers improved spatial and object recognition memory in SAMP8. The density of axons in the hippocampal CA1 was significantly increased by oligomer administration. Moreover, the administration of oligomers increased the phosphorylation of vascular endothelial growth factor receptor (VEGFR)-2 in the hippocampal CA3, hypothalamus, and choroid plexus. We speculate that memory improvement accompanied by histological changes may be induced directly in the hippocampus and indirectly in the hypothalamus and choroid plexus through VEGFR-2 signaling. In the present study, we elucidated the protective effect of oligomers against memory impairment with aging. VEGFR-2 signaling may provide a new insight into ways to protect against memory deficit in the aging brain.

## 1. Introduction

Proanthocyanidins are known as condensed tannins, members of a specific group of polyphenolic compounds, and they have been reported to exhibit powerful antioxidant activity [1,2]. Although proanthocyanidin is the most abundant dietary polyphenol, its high-level polymerization results in limited absorption *in vivo* [3]. We previously isolated oligomeric proanthocyanidins from persimmon peel, which is usually discarded even though it is rich in phenolic compounds [4]. The amount of proanthocyanidin in the peel is higher than in the rest of the fruit. It was reported that oligomeric proanthocyanidins (oligomers) isolated from persimmon peel increased the expression of silent information regulator two ortholog 1 (Sirt1), which is recognized as an essential factor in lifespan extension, in an H_2_O_2_-induced cellular senescence model. Oligomer treatment also decreased the expression level of 8-hydroxy-2'-deoxyguanosine (8-OHdG), a marker of oxidation in the model [5]. In the present study, we investigated the possibility of oligomers extending the lifespan of senescence-accelerated mouse (SAM) prone/8 (SAMP8). Since dietary restriction extends the lifespan of rodents, we compared food-restricted with oligomer-treated mice regarding longevity and behavioral characters. Moreover, we hypothesized that the oligomeric form of proanthocyanidins exerts a beneficial effect on memory dysfunction and neuroprotection in the aged brain. Using the SAMP8 model, we investigated the effect of oligomers on spatial and object recognition memory, and the densities of axons, dendrites, and synapses were observed. Furthermore, to evaluate the neuroprotective effect, vascular endothelial growth factor receptor (VEGFR)-2 as a possible modifier of motor neuron degeneration and its phosphorylation were also investigated.

## 2. Fractionation of Oligomeric Proanthocyanidins of Persimmon Fruits

**Figure 1:**
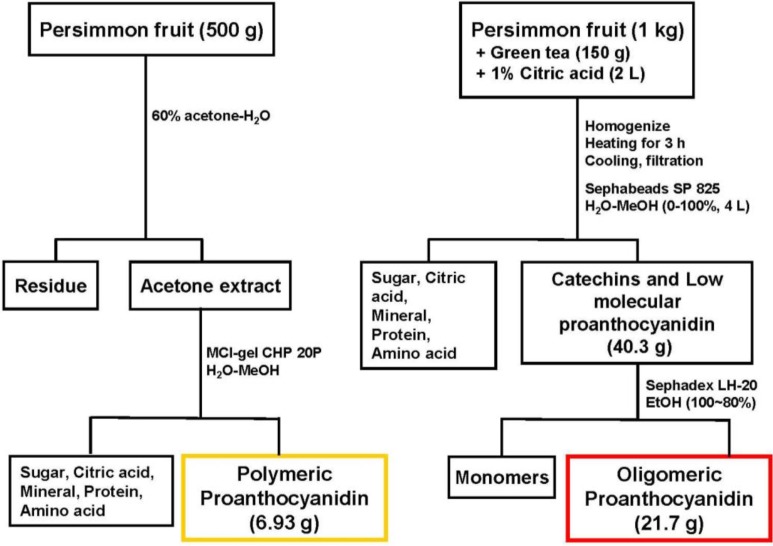
Fractionation of persimmon oligomeric proanthocyanidins.

As described previously [6], a mixture of freshly crushed persimmon fruits (unripened, 5–7 cm in diameter, 1 kg) and dried green tea leaves (150 g) in water containing 1% citric acid (2 L) was boiled for 3 h. At this stage, nucleophillic substitution at the C-4 positions of polymeric proanthocyanidins with monomeric tea catechins occurred, and, consequently, the polymeric molecules were converted into oligomers. After cooling, insoluble materials were removed by filtration, and the filtrate was directly applied to a Sephabeads SP 825 column (10 cm i.d. × 45 cm, Mitsubishi Chemical Co., Japan). Elution with water (4 L) washed out non-phenolic compounds consisting of citric acid, sugars, minerals, amino acids, *etc*. Further elution with water containing increasing amounts of methanol (0%–100% methanol, 20% stepwise elution, 4 L each) yielded a mixture of oligomeric proanthocyanidins and tea catechins (40.3 g). The mixture was subsequently subjected to Sephadex LH-20 column chromatography with ethanol. The monomeric tea catechins were eluted out with ethanol, and oligomers were yielded (21.7 g) (Figure 1). The degree of oligomeric polymerization was estimated to be 3.3 by quantitative HPLC analysis of thiol degradation products [7], while the proportions of epigallocatechin (EGC), epicatechin (EC), epigallocatechin 3-*O*-gallate (EGCg), and epicatechin 3-*O*-gallate (ECg) in oligomers were determined to be 47, 15, 31, and 6%, respectively.

## 3. Oligomeric Proanthocyanidins Extend Lifespan of SAMP8

Increased longevity is one of the most common desires of human beings. Therefore, anti-aging research is ultimately focused on lifespan extension. However, no convincing strategy based on scientific evidence has been suggested, except for dietary restriction [8]. Lifespan extension by dietary restriction has been observed over the years in many species, including rats, mice, hamsters, dogs, fish, invertebrates, and yeast. Despite these very encouraging results, clinical application is complex and limited. Regarding this point, although various dietary restriction mimetics, such as glycolytic inhibitors and antioxidants, have been suggested, scientific evidence must be accumulated to support their application [8]. For this reason, the search for novel anti-aging agents to elicit the same beneficial effects as caloric restriction without side effects and toxicity has attracted much attention.

**Figure 2:**
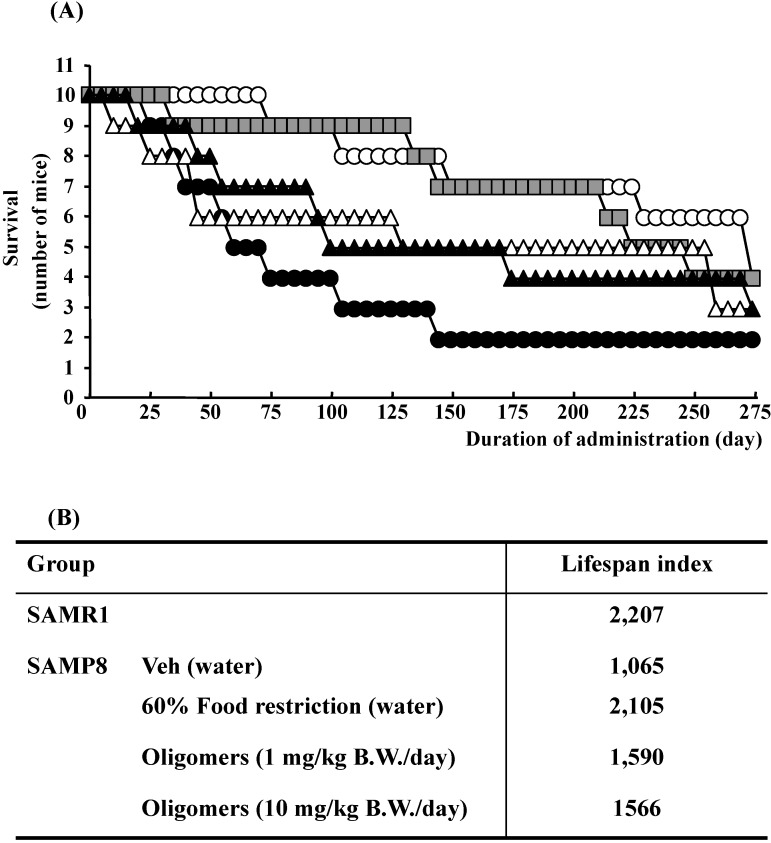
Effects of oligomers on lifespan of SAM. Forty-five or forty-six-week-old SAMP8 mice were administered vehicle (Veh, water p.o., *n* = 10), while another two groups were administered oligomers orally at doses of 1 or 10 mg/kg body weight/day (*n* = 10) using a stomach tube until death. For the remaining group of mice, the mean food intake was restricted to 60% until death (*n* = 10). SAMR1 mice (45–46 weeks old, *n* = 10) were used as a control group. (**A**) Effects of oligomers on survival of SAMP8. (**B**) Lifespan index based on survival data. Open circle: SAMR1; closed circle: SAMP8 (Veh); open triangle: SAMP8 (oligomers at 1 mg/kg B.W./day); closed triangle: SAMP8 (oligomers at 10 mg/kg B.W./day); gray square: SAMP8 (60% food restriction).

The lifespan of SAMP8 mice were significantly decreased compared to control strain SAM-resistance/1 (SAMR1, as controls), whereas, the administration of oligomers extended the lifespan, as shown in Figure 2. However, the lifespan does not extend in response to an increase in the oral dose of oligomers. The bioactivity of catechin derivatives is related to their structural phenolic groups. The increase in the level of polymerization means a rise in phenolic group contents. Previously, we demonstrated that proanthocyanidins showed strong antioxidative activities accompanying with monomeric catechin derivatives *in vitro* [9]. Many researchers have suggested that antioxidative activities are associated with a delay in the aging process and extension of the lifespan in various organisms [10]. Actually, we demonstrated that oligomers increased Sirt1 expression, a related protein of longevity extension, in a cellular senescence model [5]. Therefore, we expected oligomeric proanthocyanidins to exert a powerful activity to extend the lifespan due in part to antioxidative effects.

To elucidate the related mechanisms, the expression of Sirt1 was observed. Sir2 is an NAD^+^-dependent deacetylase implicated in the regulation of lifespan in species as diverse as yeast, worms, and flies [11]. Yeast Sir2 is a heterochromatin component that silences transcription at the silent mating loci, telomeres, and ribosomal DNA [12]. In addition, it suppresses recombination in ribosomal DNA and modulates the longevity of most organisms, including mammals [13,14]. Therefore, the enzymatic activity of Sir2 may indicate its usefulness as an effective caloric restriction mimetic [15]. Among the seven mammalian homologs of Sir2, Sirt1 is the human ortholog of yeast Sir2 and the best-characterized member of mammalian sirtuins. Recently, we showed that pretreatment with oligomers significantly increased Sirt1 expression in a cellular senescence model [5]. Therefore, in the present study, we investigated the effect of oligomers on the expression of Sirt1 in the SAM model.

**Figure 3:**
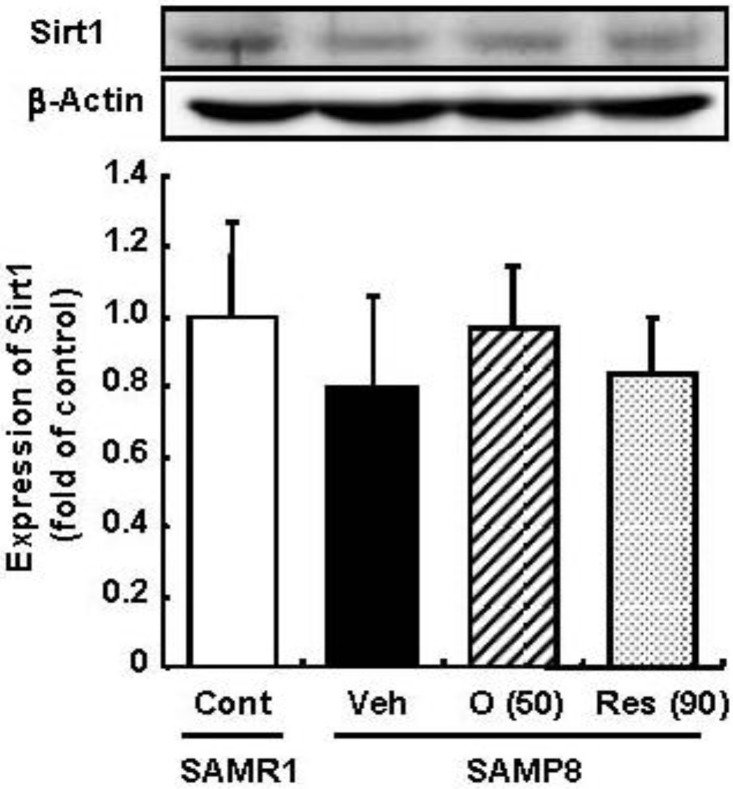
Effects of oligomers on Sirt1 expression in the brain of SAM. Forty-five-week-old SAMP8 were administered vehicle (Veh, water p.o., *n* = 5), oligomers (O (50), 50 mg/kg B.W./day, p.o., *n* = 5), or resveratrol (Res (90), 90 mmol/kg B.W./ day, p.o., *n* = 5). After 5 weeks of administration, brain lysates were immunoblotted with antibodies for Sirt1. Sirt 1 expression intensities were divided by β-actin expression. SAMR1 mice were used as a control (Cont, *n* = 4).

Resveratrol has been reported to promote the fitness and survival of simple organisms such as *Saccharomyces cerevisiae* [16,17] as well as mice fed high-calorie diets [18,19] by activation of Sirt1. Moreover, we previously clarified that oligomers increased Sirt1 expression in a cellular senescence model [5]. Therefore, the effect of oligomers on Sirt1 was compared with resveratrol *in vivo*. We expected the administration of oligomers to increase the expression and activation of Sirt1 in the brain and to slow aging-related deteriorations of SAMP8. In this study, the administration of oligomers slightly elevated Sirt1 expression in the brain of SAMP8, but not significant (Figure 3). The treatment of resveratrol did not have any effect on Sirt1.

**Figure 4:**
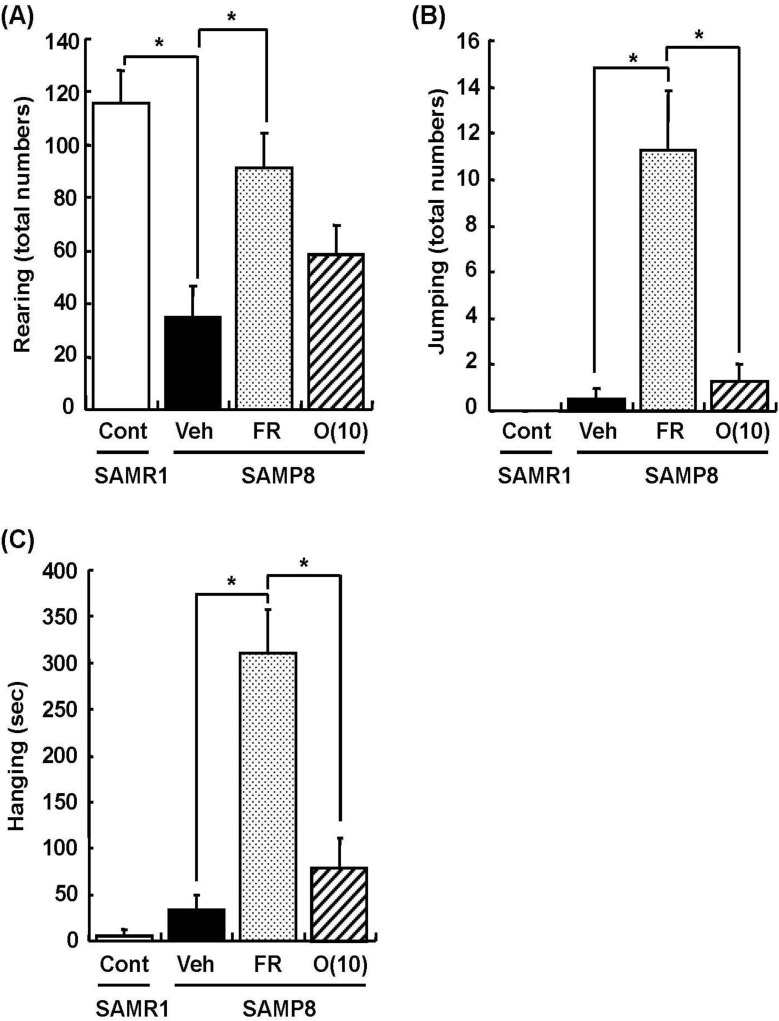
Effects of oligomers on stereotypical behaviors. Forty-five or forty-six-week-old SAMP8 mice were administered vehicle (Veh, water p.o., *n* = 4), while another two groups were administered oligomers orally at doses of 10 mg/kg body weight/day (O (10), *n* = 4) using a stomach tube until death. For the remaining group of mice, the mean food intake was restricted to 60% until death (FR, *n* = 4). SAMR1 mice (Cont, 45–46 weeks old, *n* = 4) were used as a control group. One hundred and thirty-nine days after the start of administration, actions of rearing up on the hindlimbs and jumping from the bottom of the cage were counted for 15 min (**A**, **B**). The time spent hanging from the lid was measured for 10 min (**C**). Administration was continued during the tests. ^a^ *p* = 0.0034 (Student’s *t*-test); * *p* < 0.05 (One-way ANOVA, *post-hoc* Bonferroni’s test).

**Figure 5:**
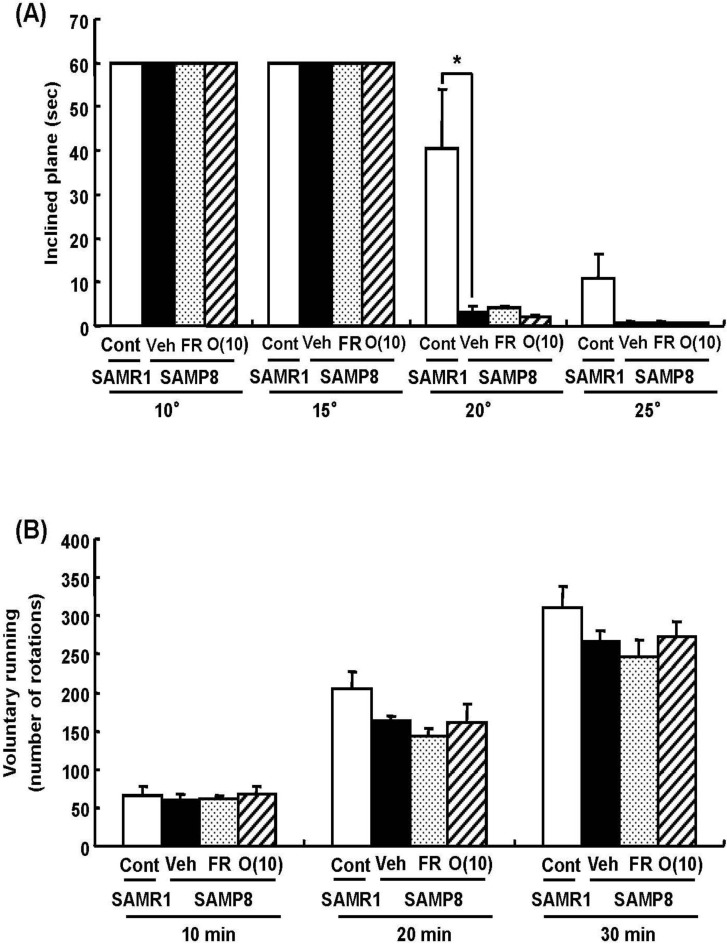
Effects of oligomers on motor function. Forty-five or six-week-old SAMP8 mice were administered vehicle (Veh, water p.o., *n* = 4), while another two groups were administered oligomers orally at doses of 10 mg/kg body weight/day (O (10), *n* = 4) using a stomach tube until death. For the remaining group of mice, the mean food intake was restricted to 60% until death (FR, *n* = 4). SAMR1 mice (Cont, 45-46 weeks old, *n* = 4) were used as a control group. One hundred and thirty-nine days after the start of administration, the time spent on the inclined surface without dropping was measured (**A**). The number of rotations measured for 30 min (**B**). Administration was continued during the tests. ^a^ *p* = 0.0159 (A: Student’s *t*-test).

Dietary restriction as an effective method for the extension of longevity has also been reported to induce stereotypical behaviors such as rearing and jumping independent of the lifespan [20]. In behavioral analyses, we showed that rearing, jumping, and hanging from the lid of the cage in 60% food-restricted SAMP8 markedly increased compared with vehicle-treated SAMP8. Surprisingly, oligomer-treated SAMP8 did not show an increase in these stereotypical behaviors (Figure 4). Moreover, in inclined plane and voluntary running tests performed to observe differences in motor function, we found no significant difference in the motor function among all SAMP8 groups (Figure 5). These results indicate that stereotypical behaviors shown in the 60% food-restricted group have no relationship with motor function. It has been reported that dietary restriction may induce anxiety-like behavior by the down-regulation of corticotrophin-releasing factor [21]. Diet-restricted rats showed stereotypy by an increase of dopamine receptor signaling. Chen *et al.* [20] demonstrated that stereotypical behaviors brought about by caloric restriction were eliminated in Sirt1-knockout mice, indicating that Sirt1 activation may cause stereotypical behaviors on dietary restriction. In our study, although the lifespan was extended by oligomers as well as 60% food restriction, mice administered oligomers did not show stereotypical behaviors, like Sirt1-knockout mice undergoing food restriction.

## 4. Oligomeric Proanthocyanidins Improve Memory and Enhance Phosphorylation of VEGFR-2 in SAMP8

SAMP8 developed age-related cognitive deficit at as early as 4 months and had a short lifespan relative to SAM-resistance/1 (SAMR1). SAMP8 show a decrease in the release of acetylcholine and noradrenaline in comparison with age-matched SAMR1 [22,23]. Many age-dependent alterations in various brain regions such as the cerebral cortex and hippocampus at an early stage in SAMP8 have been suggested as causes of memory deficit [24,25]. In the hippocampus, there was a greater increase of glial fibrillary acidic protein as an astrocyte marker in CA1-3 regions of SAMP8 compared with age-matched SAMR1, indicating enhanced reactive gliosis in aged SAMP8 [26]. Tanaka *et al.* [27] reported the severe loss of oligodendrocytes in the hippocampal CA1 of SAMP8. Moreover, neuronal loss and lower-level expressions of glial cell line-derived neurotrophic factor in the hippocampal CA1 associated with memory impairment of SAMP8 were reported [28]. Therefore, hippocampal dysfunctions of SAMP8 have been considered to be a major cause of age-dependent memory impairment. Various candidate therapeutic agents for memory dysfunction in SAMP8 have been reported, such as acetyl-L-carnitine, α-lipoic acid, and Choto-san (a herbal formula medicine), as well as caloric restriction [29,30,31,32]. In those studies, oxidative stress was focused on as a cause of memory impairment in SAMP8, although the change of oxidative stress was limited in the cerebral cortex. Additionally, neuronal morphological evaluations were insufficient in those studies.

**Figure 6:**
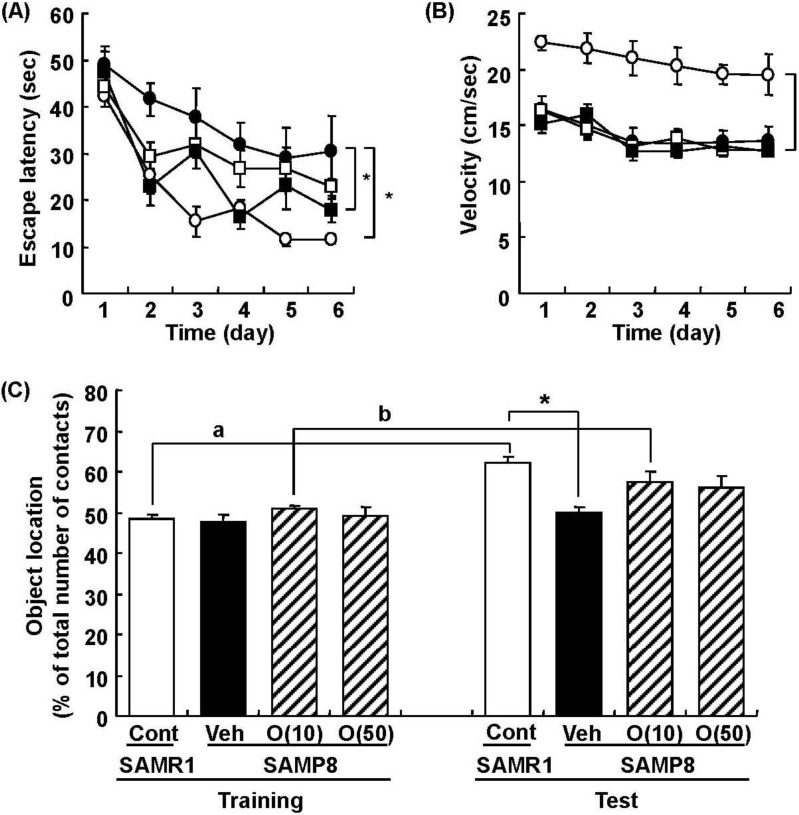
Effects of oligomers on spatial memory deficit in SAMP8. Eighteen-week-old SAMP8 were administered vehicle (Veh, water p.o., *n* = 6; closed circles) or oligomers (10 mg/kg body weight/day, p.o., *n* = 6; open squares or 100 mg/kg body weight/day, p.o., *n* = 5; closed squares) for 5 weeks. SAMR1 were used as a control (Cont, *n* = 5; open circles). Fifteen days after the start of administration, memory acquisition tests were continued for 6 days in a Morris water maze. Administration was continued during the tests. Escape latencies to a hidden platform were measured (**A**). The swimming velocities of mice in the memory acquisition test are shown (**B**). Thirty-eight-week-old SAMP8 were administered vehicle (Veh, water p.o., *n* = 7) or oligomers (O (10), 10 mg/kg body weight/day, p.o., *n* = 7 or O (50), 50 mg/kg body weight/day, p.o., *n* = 7). Age-matched SAMR1 were used as a control (Cont, *n* = 7). Twenty-eight days after administration started, an object location test was performed. The preference index was defined as the number of times a mouse made contact with any one of the objects (training session) or the moved object (test session) out of the total number of times the mouse made contact with both objects (%) (**C**). * *p* < 0.05 *vs.* Veh. (A and B: Repeated measures two-way ANOVA followed by Dunnett’s or Bonferroni’s *post-hoc* test); * *p* < 0.05 (C: One-way ANOVA followed by Bonferroni’s *post-hoc* test); ^a ^*p* = 0.0005;^ b^ *p* = 0.0213 (C: paired *t*-test).

**Figure 7:**
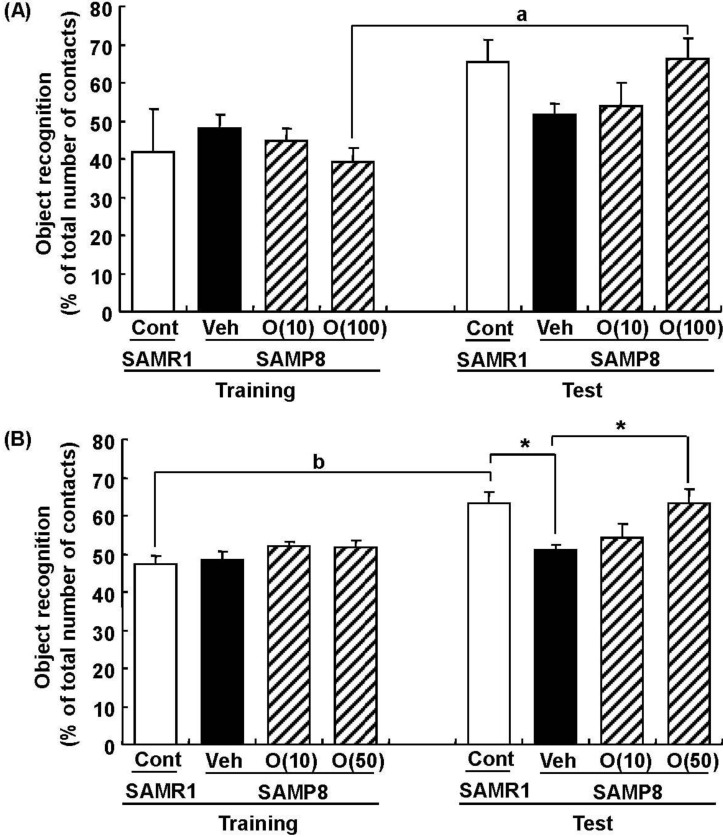
Effects of oligomers on object recognition memory deficit in SAMP8. Eighteen-week-old SAMP8 were administered vehicle (Veh, water p.o., *n* = 6) or oligomers (O (10), 10 mg/kg body weight/day, p.o., *n* = 6 or O (100), 100 mg/kg body weight/day, p.o., *n* = 5). Age-matched SAMR1 were used as a control (Cont, *n* = 5). Twenty-four days after the start of administration, a novel object recognition test was performed (**A**). Thirty-eight-week-old SAMP8 were administered vehicle (Veh, water p.o., *n* = 7) or oligomers (O (10), 10 mg/kg body weight/day, p.o., *n* = 7 or O (50), 50 mg/kg body weight/day, p.o., *n* = 7). Age-matched SAMR1 were used as a control (Cont, *n* = 7). Twenty-three days after the start of administration, a novel object recognition test was performed (**B**). The preference index was defined as the number of times a mouse made contact with any one of the objects (training session) or the novel object (test session) out of the total number of times the mouse made contact with both objects (%). * *p* < 0.05 (One-way ANOVA followed by Bonferroni’s *post-hoc* test); ^a^ *p* = 0.0174; ^b^ *p* = 0.0014 (paired *t*-test).

**Figure 8:**
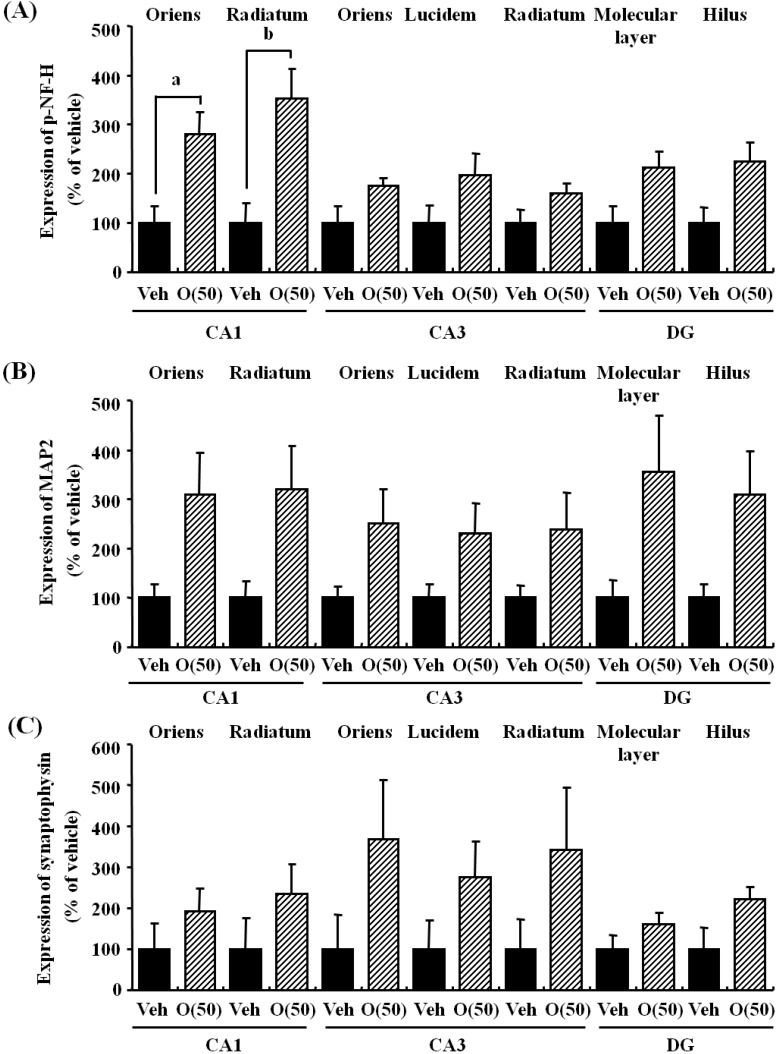
Effects of oligomers on the decrease of axons, dendrites, and synapses in the hippocampus. Fifty-nine-week-old SAMP8 were administered vehicle (Veh, water p.o., *n* = 3) or oligomers (O (50), 50 mg/kg body weight/day, p.o., *n* = 3). After seven days of administration, brain slices were immunostained with p-NF-H (**A**), MAP2 (**B**), and synaptophysin antibodies (**C**). The intensities of immuno-positive areas in the hippocampus were quantified. ^a ^*p* = 0.0243;^ b^ *p* = 0.0344 (Student’s *t*-test).

In our study, oligomers improved spatial memory and object recognition memory in SAMP8. The memory improvements seen in 18- and 38-week-old SAMP8 led to memory levels almost the same as those of SAMR1 (Figure 6 and Figure 7). To investigate the neurological changes brought about by the oral administration of oligomers, we carried out immunohistological analysis of the brain of 59-week-old SAMP8. Figure 8 shows the effect of oligomers on axons, dendrites, and synapses in the oriens and radiatum of hippocampal CA1; oriens, lucidem and radiatum of hippocampal CA3; and molecular layer and hilus of the dendate gyrus (DG). The expression levels of neurofilament-H (p-NF-H; axon marker) were increased in oligomer-administered compared with vehicle-treated mice. The expression levels of microtubule-associated proteins (MAP) 2a and 2b (MAP2; dendrite marker) and synaptophysin were slightly increased in oligomer-administered mice. In particular, the expression of p-NF-H significantly increased in the hippocampal CA1 by oligomer administration. p-NF-H is used as a marker of axons, since the phosphorylated form of NF-H is translocated into axons [33]. In the hippocampus of aged mice, fragments of degenerated axons were also increased, although reductions of neuronal numbers are small in this region [34]. Axonal termination to the spine is a necessary step for synaptogenesis. Considering synaptic losses in the hippocampal CA1 and CA3 and the parietal cortex in SAMP8 [35], as well as in the hippocampal CA1, CA3, and DG in aged rats [36], axonal regeneration is important for improving the hippocampal function. Therefore, the increased density of axons in the hippocampal CA1 was suggested to have a protective role against memory loss with aging.

**Figure 9:**
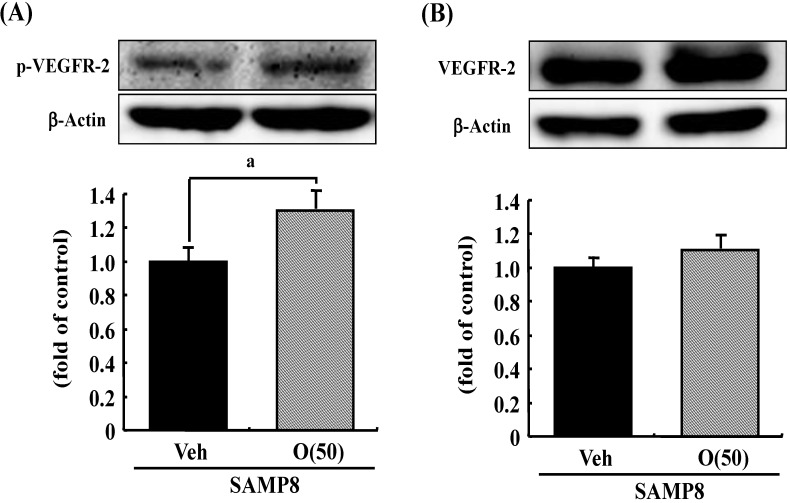
Effects of oligomers on phosphorylated VEGFR-2 (p-VEGFR-2) and VEGFR-2 expressions. Fifty-nine-week-old SAMP8 were administered vehicle (Veh, water p.o.) or oligomers (O (50), 50 mg/kg body weight/day, p.o.). After seven days of administration, brain lysates were immunoblotted with antibodies for p-VEGFR-2 (**A**) or VEGFR-2 (**B**). Expression intensities were divided by β-actin expressions to calculate ratios. ^a ^*p* = 0.0481 *vs.* O (50) (Student’s *t*-test).

**Figure 10:**
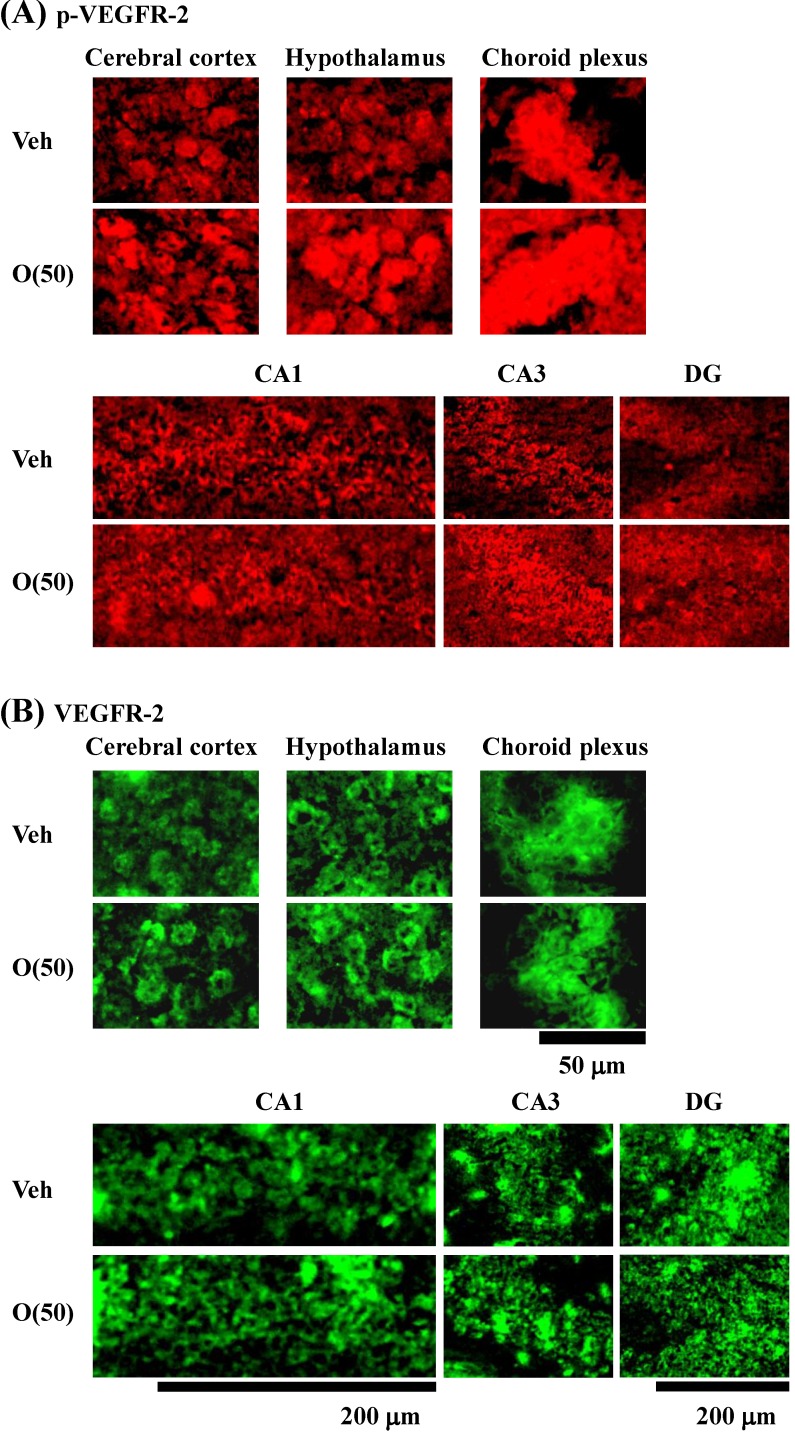
Effects of oligomers on p-VEGFR-2 and VEGFR-2 expression in various brain regions. Fifty-nine-week-old SAMP8 were administered vehicle (Veh, water p.o., *n* = 3) or oligomers (O (50), 50 mg/kg B.W./day, p.o., *n* = 3). After seven days of administration, brain slices were immunostained with p-VEGFR-2 (**A**) and VEGFR-2 (**B**) antibodies.

**Figure 11:**
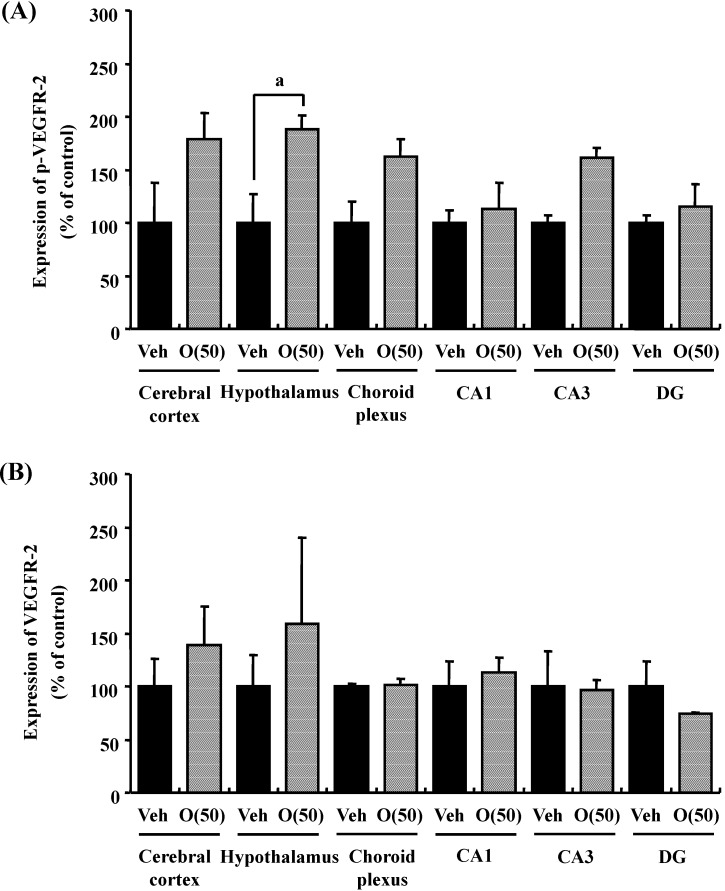
Intensities of p-VEGFR-2- (**A**) and VEGFR-2 (**B**)-positive areas were quantified in the cerebral cortex, hypothalamus and choroid plexus, and the CA1, CA3, and DG of the hippocampus. ^a^ *p* = 0.0429 *vs.* O (50) (Student’s *t*-test).

**Figure 12:**
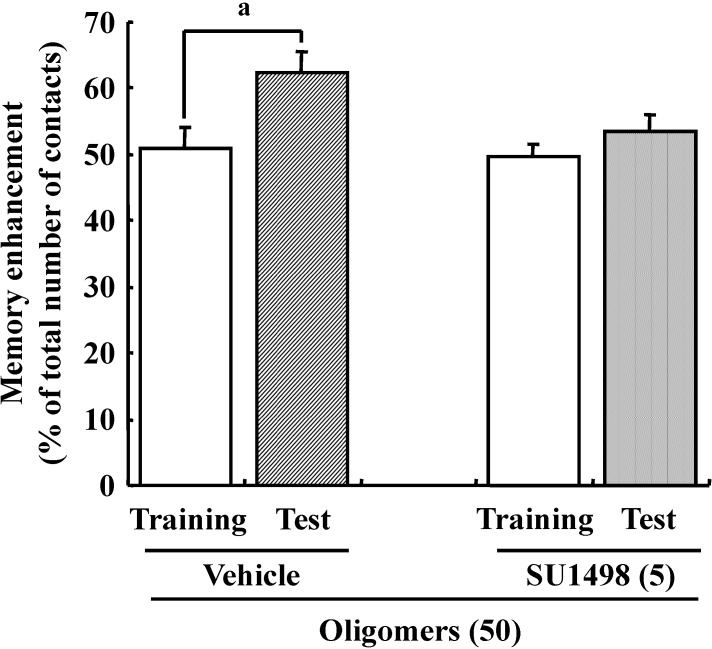
Effects of oligomers and VEGFR-2 on memory. Male ddY mice (6 weeks old) were administered oligomers (O (50), 50 mg/kg body weight/day, p.o., *n* = 4) for 7 days. Then, the vehicle (Veh, 5% DMSO in 0.9% NaCl) was injected intracerebroventricularly at 60 min after the final administration of oligomers. Five days after vehicle injection, SU1498(5 nmol/μL, solution is 5% DMSO in 0.9% NaCl) was injected intracerebroventricularly at 60 min after the final administration of oligomers. ^a^ *p* = 0.0392 (Student’s *t*-test).

Previous studies suggested that oxidative stress is a major cause of memory impairment in SAMP8. Hippocampus-specific modulation by oligomers is not explained by an anti-oxidative effect, since only the cerebral cortex is susceptible to oxidative stress in SAMP8 and not the hippocampus [37]. Therefore, to investigate target molecules by the oral administration of oligomers in the brain of SAMP8, we performed a receptor tyrosine kinase phosphorylation antibody array, and clarified that oligomer treatment increased the phosphorylation of VEGFR-2, as shown in Figure 9. In addition the effect of oligomers on the expression in various brain regions was also investigated ( Figure 10). Increased expressions of p-VEGFR-2 were observed in the cerebral cortex, hypothalamus, choroid plexus, and hippocampal CA3 layer of SAMP8 (Figure 11A). In particular, the p-VEGFR-2 level in the hypothalamus of oligomer-treated SAMP8 was significantly increased compared with that in vehicle-treated mice. On the other hand, expression levels of VEGFR-2 in an SAMP8 group administered oligomers did not show significant changes compared with vehicle-administered mice in all brain regions observed (Figure 11B). In neurons, stimulation by VEGFR-2 among protein tyrosine kinase receptors of VEGF is linked to Akt/PKB activation and neuronal protection in hypoxic preconditioning [38]. Moreover, VEGFR-2 mediated a protective effect through phosphatidylinositol-3-kinase/Akt- and mitogen-activated protein/extracellular signal-regulated kinase-signaling pathways in glutamate-induced toxicity [39]. In particular, memory enhancement in recombinant adeno-associated viral vectors expressing human VEGF-injected mice was inhibited by the injection of dominant-negative mutant VEGFR-2 [40]. This indicates that VEGF/VEGFR-2 is directly associated with neuronal signaling. VEGF also exerts indirect effects on neurons. Moreover, the topical administration of VEGF to the surface of the brain reduces the infarct size, and intraventricular VEGF enhanced the survival of newly generated neurons in the dentate gyrus and subventicular zones after focal cerebral ischemia [41]. In this study, we firstly showed that memory enhancement through oligomer treatment was eliminated by SU1498, an inhibitor of VEGFR-2 (Figure 12). Considering that VEGF-E-induced memory was also inhibited by SU1498, oligomers or their metabolites may regulate memory by the activation of VEGFR-2.

We found that the administration of oligomers increased the phosphorylation of VEGFR-2 in the hippocampal CA3 region, suggesting that oligomeric metabolites directly affect the hippocampus, like the VEGFR-2 ligand. It has been reported that Ca^2+^ influx and synaptic transmission by VEGF in the hippocampus influences the generation of long-term changes in synaptic efficacy [42]. VEGF also stimulates neurite outgrowth via Rho/ROK signaling in cerebral cortical neurons [43]. Interestingly, changes in the synapes and neurites induced by VEGF are caused by the activation of VEGFR-2 rather than VEGFR-1. Therefore, we speculated that the phosphorylation of VEGFR-2 induced by the administration of oligomers within the hippocampus may be related to an increase in the densities on neurites and synapses in the hippocampus.

The administration of oligomers increased the phosphorylation of VEGFR-2 in the hypothalamus and choroid plexus as well as the hippocampus. The hypothalamus is contained in the Papez circuit. The Papez circuit is a sensory circuit involving the thalamus, sensory cortex (especially the cingulate region), hippocampus, and mammillary body of the hypothalamus [44]. It has been reported that lesions in the Papez circuit are associated with amnesia and the impairment of recognition memory [45]. Therefore, we speculate that the hypothalamus is activated by the phosphorylation of VEGFR-2, which may affect the hippocampus through the Papez circuit.

The choroid plexus is made up of numerous villi which project into the ventricles of the brain. Each villus is composed of a single layer of epithelial cells overlying a core of connective tissues and blood capillaries [46]. The choroid plexus is involved in the most basic aspects of neural function, including maintaining the extracellular milieu of the brain by actively modulating chemical exchange between the cerebrospinal fluid and brain parenchyma, surveying the chemical and immunological status of the brain, detoxifying the brain, secreting a nutritive cocktail of polypeptides, and participating in repair processes following trauma. This diversity of functions may mean that even modest changes in the choroid plexus can have far-reaching effects [47]. Actually, a host of growth factors and other neuroprotective agents given via the cerebrospinal fluid can minimize the adverse effects of stroke on the rat hippocampus. Multiple functional failures including a decrease of cerebrospinal fluid as well as the atrophy of choroidal epithelial cells shown in normal aging as well as advanced Alzheimer’s disease indicate that the maintenance of cerebrospinal fluid through the choroid plexus may have beneficial effects against neurodegenerative diseases [48]. Moreover, it was reported that the intracerebroventicular injection of nerve growth factor or insulin-like growth factor-1 improved memory deficit and hippocampal deterioration [49,50]. Therefore, we speculate that oligomers may induce the secretion of some peptides after the phosphorylation of VEGFR-2 in the choroid plexus, and then this peptide may induce changes in the hipppocampus.

## 5. Acute Toxicity Studies

We carried out an investigation of the side effects or toxicity of oligomers. The results showed normal ranges of biochemical parameters such as alanine aminotransaminase, aspartate aminotransaminase, and blood urea nitrogen as well as changes in body and tissue weights, although the maximum concentration for oral administration was higher (500 mg/kg body weight/day) than the average dietary intake of proanthocyanidins of 58 mg/day of humans in the United States [51]. Therefore, we suggest that oligomeric proanthocyanidins are safe and novel anti-aging agents associated with life span extension.

## 6. Closing Remarks

We previously revealed that oligomers consisted of various combinations of 4 types of monomer: EGC, EC, EGCg, and ECg. Oligomers containing dimers, trimers, and tetramers of EGC, EC, EGCg, and ECg are considered to exert a stronger activity than polymers. Several researches suggest that absorption and utilization of oligomers to prevent age-dependent changes depends on their structure and/or polymerization [52,53,54]. Therefore, the next study must be elucidated that the similarities and differences in activities and functional mechanisms between oligomers and metabolites including monomers *in vivo*.

In summary, oligomer administration exerted its effect on extension of the lifespan with a tendency of increased Sirt1 expression of brain tissue and without stereotypical behavior in SAMP8. Moreover, the density of axon in the hippocampal CA1 and the phosphorylation of VEGFR-2 in the hippocampal CA3, hypothalamus, and choroid plexus were increased by oligomers, which influenced on the memory improvement directly or indirectly. Therefore oligomeric forms of proanthocyanidins could act as a potential therapeutic agent against neurodegenerative disease in the brain.

## References

[1] Dixon R.A., Xie D.Y., Sharma S.B. (2005). Proanthocyanidins—A final frontier in flavonoid research?. New Phytol..

[2] Xie D.Y., Dixon R.A. (2005). Proanthocyanidin biosynthesis—Still more questions than answers?. Phytochemistry.

[3] Manach C., Williamson G., Morand C., Scalbert A., Rémésy C. (2005). Bioavailability and bioefficacy of polyphenols in humans. I. Review of 97 bioavailability studies. Am. J. Clin. Nutr..

[4] Gorinstein S., Zachwieja Z., Folta M., Barton H., Piotrowicz J., Zemser M., Weisz M., Trakhtenberg S., Màrtìn-Belloso O. (2001). Comparative contents of dietary fiber, total phenolics, and minerals in persimmons and apples. J. Agric. Food Chem..

[5] Lee Y.A., Cho E.J., Yokozawa T. (2008). Protective Effect of persimmon (*Diospyros kaki*) peel Proanthocyanidin against oxidative damage under H_2_O_2_-induced cellular senescence. Biol. Pharm. Bull..

[6] Lee Y.A., Kim Y.J., Cho E.J., Yokozawa T. (2007). Ameliorative effects of proanthocyanidin on oxidative stress and inflammation in streptozotocin-induced diabetic rats. J. Agric. Food Chem..

[7] Tanaka T., Takahashi R., Kouno I., Nonaka G.I. (1994). Chemical evidence for the de-astringency (insolubilization of tannins) of persimmon fruit. J. Chem. Soc. Perkin Trans..

[8] Roth G.S., Lane M.A., Ingram D.K. (2005). Caloric restriction mimetics: The next phase. Ann. N. Y. Acad. Sci..

[9] Nakagawa T., Yokozawa T. (2002). Direct scavenging of nitric oxide and superoxide by green tea. Food Chem. Toxicol..

[10] Finkel T., Holbrook N.J. (2000). Oxidants, oxidative stress and the biology of ageing. Nature.

[11] Sasaki T., Maier B., Koclega K.D., Chruszcz M., Gluba W., Stukenberg P.T., Minor W., Scrable H. (2008). Phosphorylation regulates SIRT1 function. PLoS One.

[12] Imai S., Armstrong C.M., Kaeberlein M., Guarente L. (2000). Transcriptional silencing and longevity protein Sir2 is an NAD-dependent histone deacetylase. Nature.

[13] Guarente L. (2000). Sir2 links chromatin silencing, metabolism, and aging. Genes Dev..

[14] Michan S., Sinclair D. (2007). Sirtuins in mammals: Insights into their biological function. Biochem. J..

[15] Chen D., Guarente L. (2007). SIR2: A potential target for calorie restriction mimetics. Trends Mol. Med..

[16] Howitz K.T., Bitterman K.J., Cohen H.Y., Lamming D.W., Lavu S., Wood J.G., Zipkin R.E., Chung P., Kisielewski A., Zhang L.L., Scherer B., Sinclair D.A. (2003). Small molecule activators of sirtuins extend *Saccharomyces cerevisiae* lifespan. Nature.

[17] Wood J.G., Rogina B., Lavu S., Howitz K., Helfand S.L., Tatar M., Sinclair D. (2004). Sirtuin activators mimic caloric restriction and delay ageing in metazoans. Nature.

[18] Baur J.A., Pearson K.J., Price N.L., Jamieson H.A., Lerin C., Kalra A., Prabhu V.V., Allard J.S., Lopez-Lluch G., Lewis K. (2006). Resveratrol improves health and survival of mice on a high-calorie diet. Nature.

[19] Lagouge M., Argmann C., Gerhart-Hines Z., Meziane H., Lerin C., Daussin F., Messadeq N., Milne J., Lambert P., Elliott P. (2006). Resveratrol improves mitochondrial function and protects against metabolic disease by activating SIRT1 and PGC-1α. Cell.

[20] Chen D., Steele A.D., Lindquist S., Guarente L. (2005). Increase in activity during calorie restriction requires Sirt1. Science.

[21] Levay E.A., Govic A., Penman J., Paolini A.G., Kent S. (2007). Effects of adult-onset calorie restriction on anxiety-like behavior in rats. Physiol. Behav..

[22] Zhao X.H., Nomura Y. (1990). Age-related changes in uptake and release on L-[^3^H]noradrenaline in brain slices of senescence accelerated mouse. Int. J. Dev. Neurosci..

[23] Zhao X.H., Kitamura Y., Nomura Y. (1992). Age-related changes in NMDA-induced [^3^H]acetylcholine release from brain slices of senescence-accelerated mouse. Int. J. Dev. Neurosci..

[24] Kawamata T., Akiguchi I., Maeda K., Tanaka C., Higuchi K., Hosokawa M., Takeda T. (1998). Age-related changes in the brains of senescence-accelerated mice (SAM): Association with glial and endothelial reactions. Microsc. Res. Tech..

[25] Sureda F.X., Gutierrez-Cuesta J., Romeu M., Mulero M., Canudas A.M., Camins A., Mallol J., Pallàs M. (2006). Changes in oxidative stress parameters and neurodegeneration markers in the brain of the senescence-accelerated mice SAMP-8. Exp. Gerontol..

[26] Wu Y., Zhang A.Q., Yew D.T. (2005). Age related changes of various markers of astrocytes in senescence-accelerated mice hippocampus. Neurochem. Int..

[27] Tanaka J., Okuma Y., Tomobe K., Nomura Y. (2005). The age-related degeneration of oligodendrocytes in the hippocampus of the senescence-accelerated mouse (SAM) P8: A quantitative immunohistochemical study. Biol. Pharm. Bull..

[28] Miyazaki H., Okuma Y., Nomura J., Nagashima K., Nomura Y. (2003). Age-related alterations in the expression of glial cell line-derived neurotrophic factor in the senescence-accelerated mouse brain. J. Pharmacol. Sci..

[29] Yasui F., Matsugo S., Ishibashi M., Kajita T., Ezashi Y., Oomura Y., Kojo S., Sasaki K. (2002). Effects of chronic acetyl-L-carnitine treatment on brain lipid hydroperoxide level and passive avoidance learning in senescence-accelerated mice. Neurosci. Lett..

[30] Farr S.A., Poon H.F., Dogrukol-Ak D., Drake J., Banks W.A., Eyerman E., Butterfield D.A., Morley J.E. (2003). The antioxidants alpha-lipoic acid and N-acetylcysteine reverse memory impairment and brain oxidative stress in aged SAMP8 mice. J. Neurochem..

[31] Komatsu T., Chiba T., Yamaza H., Yamashita K., Shimada A., Hoshiyama Y., Henmi T., Ohtani H., Higami Y., de Cabo R. (2008). Manipulation of caloric content but not diet composition, attenuates the deficit in learning and memory of senescence-accelerated mouse strain P8. Exp. Gerontol..

[32] Mizushima Y., Kan S., Yoshida S., Irie Y., Urata Y. (2003). Effect of Choto-san, a Kampo medicine, on impairment of passive avoidance performance in senescence accelerated mouse (SAM). Phytother. Res..

[33] Dahl D., Labkovsky B., Bignami A. (1988). Neurofilament phosphorylation in axons and perikarya: Immunofluorescence study of the rat spinal cord and dorsal root ganglia with monoclonal antibodies. J. Comp. Neurol..

[34] von Bohlen und Halbach O., Unsicker K. (2002). Morphological alterations in the amygdala and hippocampus of mice during ageing. Eur. J. Neurosci..

[35] Yamamoto T., Hirayama A. (2001). Effects of soft-diet feeding on synaptic density in the hippocampus and parietal cortex of senescence-accelerated mice. Brain Res..

[36] Smith T.D., Adams M.M., Gallagher M., Morrison J.H., Rapp P.R. (2000). Circuit-specific alterations in hippocampal synaptophysin immunoreactivity predict spatial learning impairment in aged rats. J. Neurosci..

[37] Sato E., Kurokawa T., Oda N., Ishibashi S. (1996). Early appearance of abnormality of microperoxisomal enzymes in the cerebral cortex of senescence-accelerated mouse. Mech. Ageing Dev..

[38] Wick A., Wick W., Waltenberger J., Weller M., Dichgans J., Schulz J.B. (2002). Neuroprotection by hypoxic preconditioning requires sequential activation of vascular endothelial growth factor receptor and Akt. J. Neurosci..

[39] Matsuzaki H., Tamatani M., Yamaguchi A., Namikawa K., Kiyama H., Vitek M.P., Mitsuda N., Tohyama M. (2001). Vascular endothelial growth factor rescues hippocampal neurons from glutamate-induced toxicity: Signal transduction cascades. FASEB J..

[40] Cao L., Jiao X., Zuzga D.S., Liu Y., Fong D.M., Young D., During M.J. (2004). VEGF links hippocampal activity with neurogenesis, learning and memory. Nat. Genet..

[41] Sun Y., Jin K., Xie L., Childs J., Mao X.O., Logvinova A., Greenberg D.A. (2003). VEGF-induced neuroprotection, neurogenesis, and angiogenesis after focal cerebral ischemia. J. Clin. Invest..

[42] Kim B.W., Choi M., Kim Y.S., Park H., Lee H.R., Yun C.O., Kim E.J., Choi J.S., Kim S., Rhim H., Kaang B.K., Son H. (2008). Vascular endothelial growth factor (VEGF) signaling regulates hippocampal neurons by elevation of intracellular calcium and activation of calcium/calmodulin protein kinase II and mammalian target of rapamycin. Cell Signal..

[43] Jin K., Mao X.O., Greenberg D.A. (2006). Vascular endothelial growth factor stimulates neurite outgrowth from cerebral cortical neurons via Rho kinase signaling. J. Neurobiol..

[44] Dalgleish T. (2004). The emotional brain. Nat. Rev. Neurosci..

[45] Aggleton J.P., Shaw C. (1996). Amnesia and recognition memory: A re-analysis of psychometric data. Neuropsychologia.

[46] Brown P.D., Davies S.L., Speake T., Millar I.D. (2004). Molecular mechanisms of cerebrospinal fluid production. Neuroscience.

[47] Emerich D.F., Skinner S.J., Borlongan C.V., Vasconcellos A.V., Thanos C.G. (2005). The choroid plexus in the rise, fall and repair of the brain. Bioessays.

[48] Johanson C.E., Duncan J.A., Stopa E.G., Baird A. (2005). Enhanced prospects for drug delivery and brain targeting by the choroid plexus-CSF route. Pharm. Res..

[49] Jakubowska-Doğru E., Gümüşbaş U. (2005). Chronic intracerebroventricular NGF administration improves working memory in young adult memory deficient rats. Neurosci. Lett..

[50] Shi L., Linville M.C., Tucker E.W., Sonntag W.E., Brunso-Bechtold J.K. (2005). Differential effects of aging and insulin-like growth factor-1 on synapses in CA1 of rat hippocampus. Cereb. Cortex.

[51] Erdman J.W., Balentine D., Arab L., Beecher G., Dwyer J.T., Folts J., Harnly J., Hollman P., Keen C.L., Mazza G. (2007). Flavonoids and heart health: Proceedings of the ILSI North America Flavonoids Workshop, May 31-June 1, 2005, Washington, DC. J. Nutr..

[52] De Boer V.C., de Goffau M.C., Arts I.C., Hollman P.C., Keijer J. (2006). SIRT1 stimulation by polyphenols is affected by their stability and metabolism. Mech. Ageing Dev..

[53] Bagchi D., Bagchi M., Stohs S.J., Das D.K., Ray S.D., Kuszynski C.A., Joshi S.S., Pruess H.G. (2000). Free radicals and grape seed proanthocyanidin extract: Importance in human health and disease prevention. Toxicology.

[54] Weinreb O., Mandel S., Amit T., Youdim M.B. (2004). Neurological mechanisms of green tea polyphenols in Alzheimer’s and Parkinson’s diseases. J. Nutr. Biochem..

